# Web GIS in practice IX: a demonstration of geospatial visual analytics using Microsoft Live Labs Pivot technology and WHO mortality data

**DOI:** 10.1186/1476-072X-10-19

**Published:** 2011-03-16

**Authors:** Maged N Kamel Boulos, Teeradache Viangteeravat, Matthew N Anyanwu, Venkateswara Ra Nagisetty, Emin Kuscu

**Affiliations:** 1 Faculty of Health, University of Plymouth, Drake Circus, Plymouth, Devon, PL4 8AA, UK; 2 Clinical and Translational Science Institute, University of Tennessee Health Science Center, Memphis, TN 38163, USA

## Abstract

The goal of visual analytics is to facilitate the discourse between the user and the data by providing dynamic displays and versatile visual interaction opportunities with the data that can support analytical reasoning and the exploration of data from multiple user-customisable aspects. This paper introduces geospatial visual analytics, a specialised subtype of visual analytics, and provides pointers to a number of learning resources about the subject, as well as some examples of human health, surveillance, emergency management and epidemiology-related geospatial visual analytics applications and examples of free software tools that readers can experiment with, such as Google Public Data Explorer. The authors also present a practical demonstration of geospatial visual analytics using partial data for 35 countries from a publicly available World Health Organization (WHO) mortality dataset and Microsoft Live Labs Pivot technology, a free, general purpose visual analytics tool that offers a fresh way to visually browse and arrange massive amounts of data and images online and also supports geographic and temporal classifications of datasets featuring geospatial and temporal components. Interested readers can download a Zip archive (included with the manuscript as an additional file) containing all files, modules and library functions used to deploy the WHO mortality data Pivot collection described in this paper.

## Background

### What is visual analytics?

Visual analytics is an emerging area of research and practice aimed at leveraging the excellent capabilities of humans in terms of visual information exploration. Thanks to the enormous increase in the processing power of computers and their graphics handling capabilities, we are now able to implement extremely powerful visual and interactive knowledge discovery environments [1] that can empower individual researchers or groups of researchers to make well-informed decisions in complex situations [2]. The goal of visual analytics is to make the processes of data elaboration, information gathering and knowledge generation transparent to tool users. Visual analytics is an enhancement of the information visualisation concept and can be seen as an integrated approach combining visualisation, human factors and data analysis [3].

The basic idea of visual analytics is to visually represent the information, allowing the human to directly interact with the underpinning data to gain insight, draw conclusions, and ultimately make better decisions. The synergic integration between computation, visual representation, and interactive thinking supports intensive analysis by harnessing the human visual system to support the process of sense-making, in which information is collected, organised, and analysed to generate knowledge and plan actions. The goal is not only to permit users to detect expected events, such as might be predicted by models, but also to help users discover the unexpected--the surprising anomalies, changes, patterns, and relationships that are then examined and assessed to develop new insights [4].

Visual analytics is an inherently multi-disciplinary field [5-7] that aims to combine the methods and strengths of various research areas, including human-computer interaction (HCI) and usability engineering, cognitive and perceptual science, decision science, information visualisation, scientific visualisation, geospatial visualisation, databases, data mining, statistics, knowledge discovery, data management and knowledge representation, geospatial analytics, and graphics and rendering, among others.

Visual analytics comprises several areas spanning analytical reasoning techniques (to enable users to assess, plan, and make decisions), visual representations and interaction techniques (to take advantage of the human eye's broad bandwidth pathway into the mind; to allow users to see, explore, and understand large amounts of information at once), data representations and transformations (that convert all types of conflicting and dynamic data into visual and analytical representations), and techniques to support production, presentation, and dissemination of the results.

The main advantages of visual data exploration over automatic data mining techniques that use statistics or machine learning are [8]:

- Visual analytics can easily deal with highly heterogeneous and noisy data; it is intuitive and requires no understanding of complex mathematical or statistical algorithms or parameters by its operators and it is invaluable when little is known about the data and the exploration goals are vague; and

- Visual analytics can tackle hard analytical problems where neither the machine nor the human alone can efficiently and effectively find a solution.

### Geospatial visual analytics as a specialised subtype of visual analytics: resources and examples

Geospatial visual analytics is an emerging multidisciplinary area which supports spatio-temporal analytical reasoning and decision-making through interactive visual interfaces (such as maps and other visual artefacts) that are linked to computational methods [9,10]. A good online resource and starting point to learn about geospatial visual analytics is GeoAnalytics.net [11], a portal run by the Commission on GeoVisualization of the International Cartographic Association. Gennady and Natalia Andrienko also offer a comprehensive 114-slide tutorial that can be downloaded at [12].

**Figure 1:**
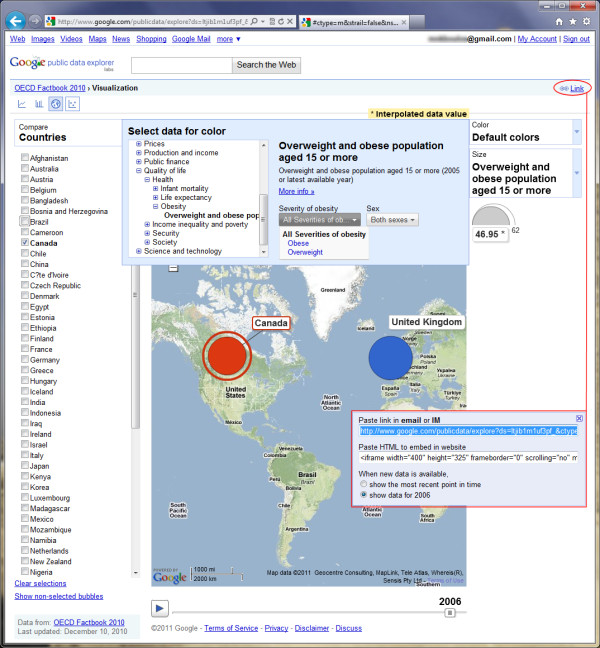
**Screenshot of Google Public Data Explorer showing some of its interface features and options**. Obesity data from OECD Factbook 2010 are being explored in this screenshot example. The user has selected two countries, Canada and UK, for comparison on a time-animated (years) map chart. Google Public Data Explorer allows custom analytical views and specific exploration results to be easily shared with others, e.g., via e-mail, and embedded in user's own Web sites.

Some examples of human health, surveillance/emergency management and epidemiology-related geospatial visual analytics applications can be found in [13-18]. Livnat and colleagues [16,17] describe Epinome [19] (a portmanteau (or blending) of 'Epidemic' and 'Panorama'), a Web-based data visualisation system for infectious disease surveillance, management and control that features movable timelines, choropleth mapping, line-list querying, in addition to other tools for interactively aggregating and stratifying data. The GeoVISTA Center at Pennsylvania State University, USA, offers a number of software applications and tools that can be used in various geospatial visual analytics scenarios [20]. Another tool of interest, Gapminder [21], allows users to explore time series of development statistics for all countries. Readers interested in creating their own Gapminder-like bubble graphs can use a free Google Gadget called Motion Chart to do so [22]. Google also has its own powerful visualisation tool (Google Public Data Explorer [23]) for exploring, visualising and sharing data in a Gapminder-like manner. Many datasets from a number of data providers such as World Bank, EuroStat, OECD (Organisation for Economic Co-operation and Development) and US CDC (Centers for Disease Control and Prevention) are currently available to explore in Google Public Data Explorer, including some sets on topics that are directly human health-related, e.g., infectious disease outbreaks, sexually transmitted diseases in the US, mortality in the US, and cancer cases in the US [24] (Figure 1). Moreover, users are able to upload their own datasets for visualisation and exploration in the same interactive manner, using a wide range of static and animated (to show change over time) line, bar, map and bubble charts, coupled with many user-selectable data classification and comparison options [25].

In this paper, we demonstrate a basic geospatial visual analytics exercise using Microsoft Live Labs Pivot technology [26], a free, general purpose visual analytics tool that offers a fresh way to visually browse and arrange massive amounts of data (and images, e.g., [27]) online. Microsoft Pivot enables powerful visual zooming in and out of Web databases and the discovery of new patterns and relations in them that would otherwise be invisible in standard Web browsing of large datasets [28]. It can also be used for geographic classifications, if the explored dataset includes a geospatial component, as we are going to show in this article.

## Microsoft Pivot technology demonstration using a publicly available WHO mortality dataset

Microsoft Pivot is a data visualisation technology that can be used to easily interact with, analyse, filter, classify and cluster large amounts of image data [29]. Pivot builds on Silverlight [30] and Deep Zoom [31] technologies, both also by Microsoft. Pivot can be created and deployed as a standalone tool using Microsoft Windows 7, Vista, or XP [29,32,33]. It can also be created using Microsoft Windows and deployed in a Web-enabled environment using most Web servers such as Apache, IIS (Microsoft Internet Information Services), etc. In this tutorial we will show how to create and deploy a Pivot project (also known as Pivot collection) using Microsoft Windows and a Web-enabled environment running a Linux (CentOS 5) operating system and Apache Web server.

### Pivot collection creation methods

**Figure 2:**
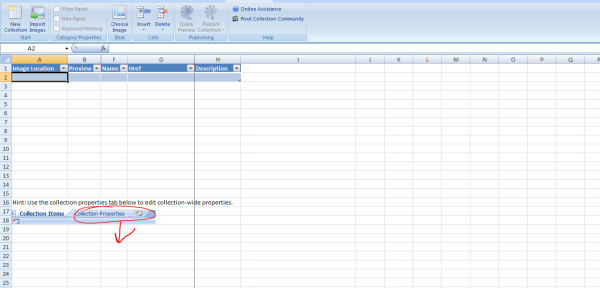
**Microsoft Excel plug-in for Pivot collections**. Screenshot showing a new spreadsheet for Pivot collection tool using the free Excel add-in for Pivot collections from Microsoft [34].

Pivot collections can be created by either automated or manual processes. In this tutorial, we will focus on the manual process using the Excel plug-in for Pivot collections that can be freely downloaded at [34]. The manual process is both simple and easy to implement. Below are some notes about the automated and manual methods for creating Pivot collections:

- **Automated process**: In this method, a computer program is developed that automates the process of creating Pivot collection images by making use of already existing libraries and modules. Common modules and libraries include, but are not limited to:

- Python Imaging Library (PIL) [35]: Version 1.1.6 or later is used in processing, creating and formatting images in various formats (JPEG--Joint Photographic Experts Group, PNG--Portable Network Graphics, etc.) for different zoom levels to produce the required visualisation; and

- Python Deep Zoom Tools: Python has a Deep Zoom library, version 0.1.0 or later [36], that is used for converting and subdividing images into various zoom levels and tiles (DZI--Deep Zoom Image file format or 'tiled image pyramid' [37]) in order to produce multi-scale zoomable high-resolution images.

- **Manual process**: In this process, a Microsoft Excel (2007 or later) spreadsheet is used to create a Pivot collection by means of an Excel add-in for Pivot collections [34]. The user does not need to write a single line of code with this method, as all the required code is already written and built into the Excel add-in for Pivot collections. The Excel plug-in acts like a collection tool for Pivot technology, in which data are represented in an Excel spreadsheet-like form using all the features of Excel (Figure 2). The data collection is then exported to a format that can be read by the PivotViewer [26]. Other requirements for the manual process include:

- Microsoft Silverlight [30]: Used in developing and creating the Pivot platform for standalone and Web-enabled applications; and

- PivotViewer [26,38]: The PivotViewer Silverlight control is included in the 'PivotViewer Collection Tool for Microsoft Excel' (the above mentioned Excel plug-in [34]). It is used for presenting Pivot collections (data and images) on demand and for visualising Pivot collections as a group.

### Essential components of a deployed Microsoft Pivot collection

**Figure 3:**
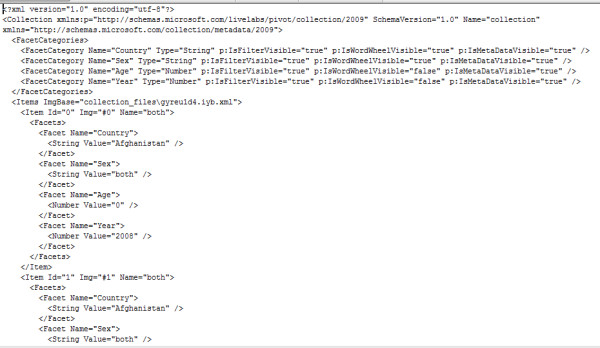
**A snippet from the collection.cxml file used in the authors' Microsoft Pivot WHO mortality data demonstration**. The complete collection.cxml file used in this demonstration can be found in the 'Additional file 1' archive.

**Figure 4:**
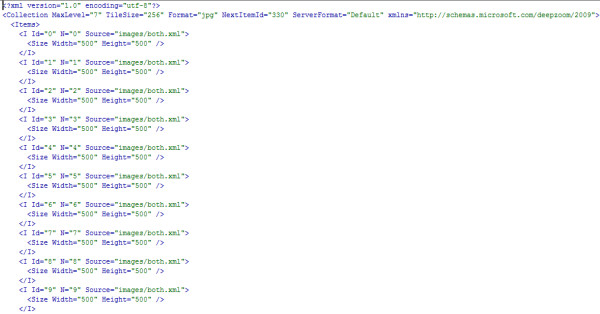
**A snippet from the collection.xml file used in the authors' Microsoft Pivot WHO mortality data demonstration**. The complete collection.xml file used in this demonstration can be found in the 'Additional file 1' archive.

**Figure 5:**
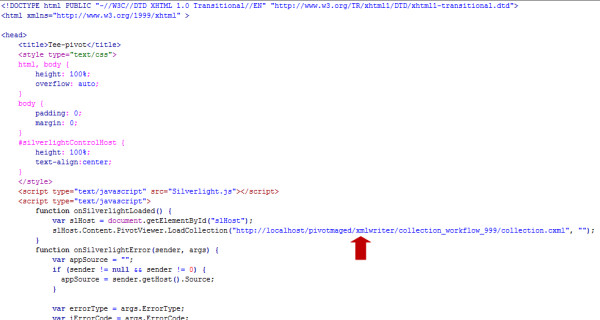
**A snippet from the collection.html file used in the authors' Microsoft Pivot WHO mortality data demonstration**. The URL (Universal Resource Locator) of the associated collection.cxml file is marked by a red arrow. The complete collection.html file used in this demonstration can be found in the 'Additional file 1' archive.

Irrespective of the Pivot collection creation method used, there are some basic modules/libraries provided by Microsoft Live Labs Pivot that are needed to complete the creation and deployment of Pivot collections, namely:

- **PivotSimpleDemo.xap**: This module/library is provided by Microsoft Live Labs. It is in compiled Silverlight application file format (actually a renamed .zip archive that contains all the files necessary for the application). When run in a Web browser, it produces a graphical user interface (GUI) for the Pivot collection.

- **Silverlight.js**: This is a JavaScript that is used to create an instance of Microsoft Silverlight and enable its browser capability. It can be downloaded at [39].

- **Collection_files**: This is a directory generated by the Pivot Collection Tool when the collection (images and data) is published using the Excel plug-in for Pivot collections. It contains the files (.cxml, .xml and .html) and images in various Deep Zoom levels necessary to launch and deploy the Pivot collection.

- **collection.cxml**: This is a file created when the Pivot collection is published using the Excel plug-in for Pivot collections. It can be given any allowable name, but the extension must always be .cxml (Collection XML--Extensible Markup Language). It can also be created using the automated process of Pivot collection generation. It contains a set of rules that describe the data sources in the collection and also a format of displaying the collection for full visualization (Figure 3).

- **collection.xml**: This file is also created automatically when Pivot collections are published using the Excel add-in tool or the automated generation method. It can have any other allowable name, but the extension must always be .xml (Extensible Markup Language). It provides unique identifications for the images in the collection at various zoom levels. It also declares their size in terms of image width and height (Figure 4).

- **collection.html**: This file is provided by Microsoft. It can assume any name but the extension is always .html (HyperText Markup Language file). It contains a reference to the Silverlight.js script, as well as the location or URL (Universal Resource Locator) of the collection.cxml file on the server used to publish the collection (marked by a red arrow in Figure 5). The collection.html file is useful when the Pivot collection is to be deployed on a Web server, as it provides the client browser capability, which Silverlight uses to display the Pivot collection in a visual form.

- **Deep Zoom Images**: These are the images at various zoom levels created with the Excel plug-in for Pivot collections or automatically using the Python Deep Zoom Tools and Python Imaging Library. The images are in DZI format and are stored in a subfolder ([images]) of the [collection_files] directory.

### Creation and deployment of a Pivot collection with the PivotViewer Collection Tool (add-in) for Microsoft Excel using a publicly available WHO mortality dataset (1990-2008)

In this demonstration, we used a sample of World Health Organization (WHO) mortality data, downloadable at [40]. The data provide mortality statistics and causes of death by country (WHO member countries), sex, age, and year. We used the following sample of data in creating the Pivot collection for this demonstration (the total number of records in the collection data sample is 984 out of 38215 records in the source WHO sheet):

- **Country**: Sample data records from 35 WHO member countries were used. The included WHO member countries are Afghanistan, Albania, Algeria, Andorra, Angola, Barbados, Belarus, Belgium, Belize, Benin, Bhutan, Cameroon, Canada, Cape Verde, Chile, China, Denmark, Djibouti, Dominica, Dominican Republic, Ecuador, Egypt, El Salvador, Equatorial Guinea, Nigeria, United Kingdom, United Republic of Tanzania, United State of America, Uruguay, Uzbekistan, Vanuatu, Venezuela, Yemen, Zambia, and Zimbabwe. Please note that only a subset of data from each country (subsets of country records and of fields in sampled records) is used in this demonstration and not the whole data for each country. Consequently, the current technology demonstrator [41] should not be used to perform any serious country analyses or comparisons, or to draw any mortality data-related scientific conclusions.

- **Age**: The age range of the population in the sample is 0-100 (0, 1, 5, 10, ..., 95, 100).

- **Sex**: Males (represented by blue man icon image), females (represented by pink woman icon image), and both (both sexes; represented by black man-and-woman-together icon image). Developers need to carefully decide on an appropriate unit value for each icon image instance (the numeric value represented by one icon or how many cases each icon will represent, e.g., one case, 10 cases, 100 cases, etc.), depending on the nature, distribution, and value range of the underpinning data.

- **Year**: The years covered by the data are 1990, 2000, and 2008.

**Figure 6:**
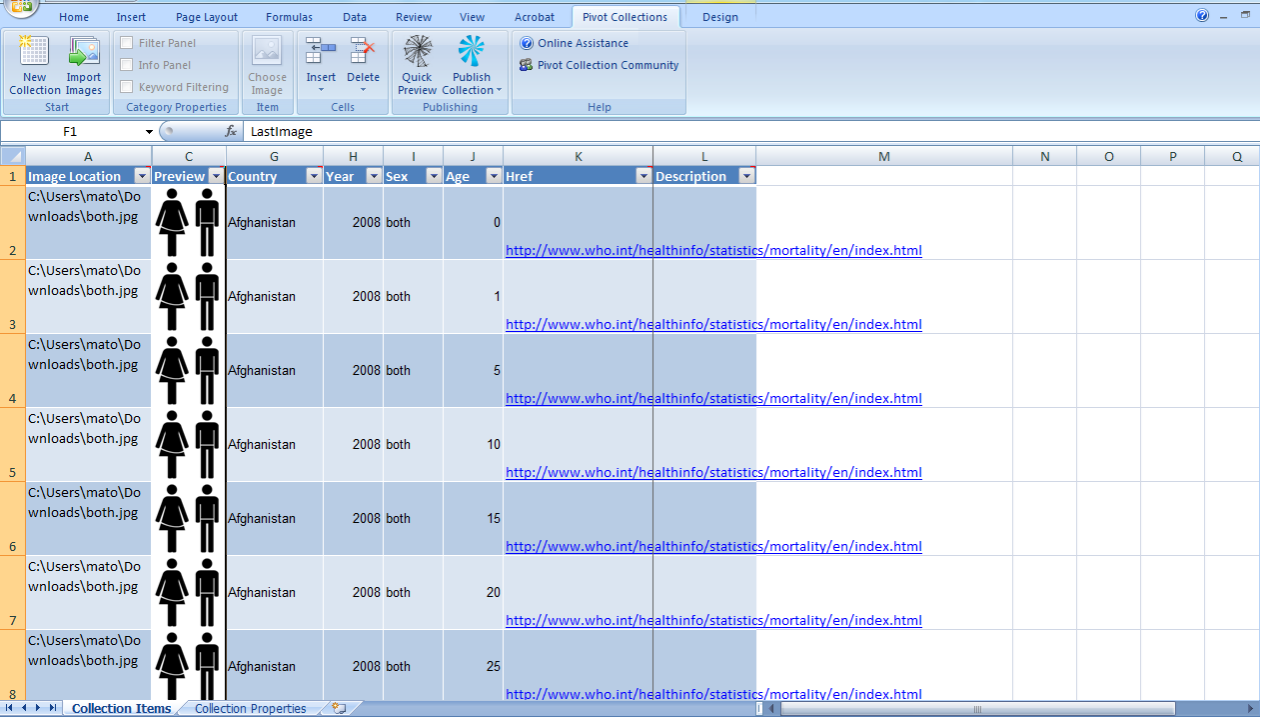
**Pivot worksheet populated with a sample of WHO mortality data in Microsoft Excel**. Images used in this collection are a blue man icon for males records, a pink woman icon for females records, and a black man-and-woman-together icon for records of both sexes.

The following steps should be followed in order to create the demonstrator Pivot collection using the WHO mortality data:

- **Step 1**: Download WHO mortality data at [40] and select the sample dataset to be used.

- **Step 2**: Install the Excel add-in for Pivot collections [34], Microsoft Silverlight [30], and PivotViewer Control [26].

- **Step 3**: Download Silverlight.js [39], and prepare PivotSimpleDemo.xap and collection.html (included in 'Additional file 1').

- **Step 4**: Create a project folder, name it [demodata] (could also be any other name), and ensure that PivotSimpleDemo.xap, Silverlight.js and collection.html are all placed in it.

- **Step 5**: Open the WHO sample data as an Excel spreadsheet.

- **Step 6**: Click on the Pivot Collection tab of the Excel workbook, then click on the New Collection tab to start a new Pivot collection workbook (the new workbook will look similar to the one shown in Figure 2).

- **Step 7**: The Pivot worksheet (Figures 2 and 6) is made up of the following columns:

- *Image Location*: This is the directory/file location of the images. Use the Choose Image Item of the Pivot workbook to locate where the images are stored;

- *Preview*: shows a preview of the images in Image Location column;

- *Name*: One of the columns that will accommodate the sample data source. Use the Insert function of the Pivot workbook to insert more columns that are needed and name the columns as required. The extra columns are the same as the Excel spreadsheet columns (*Country*, *Year*, *Sex*, *Age*);

- *Href*: Used to provide a hyperlink to data sources or image locations (can be left blank); and

- *Description*: This column is used to describe the images of data sources in the collection (can be left blank).

- **Step 8**: After adding and populating the extra columns, image locations, and Href, the Pivot worksheet will look similar to the one in Figure 6.

- **Step 9**: Publish the collection by saving the Pivot worksheet in Figure 6. Click on the Quick Preview to have a quick view visualisation of the data classification using Pivot. Figure 7 shows the Quick Preview of the Pivot collection. Note that this quick view does not display the collection images. Deploy and publish the Pivot collection in your chosen directory (e.g., [demodata]) by clicking Publish Collection tab in the Pivot worksheet. The publishing of the collection will create the necessary .cxml, and .xml files. It will also generate the images for various zoom levels and save them in a subfolder named [images]. The pivot collection will then open in a local Web browser window for viewing using the Pivot browser. Navigate through the data and images to ensure that the collection is correctly published (Figure 8).

**Figure 7:**
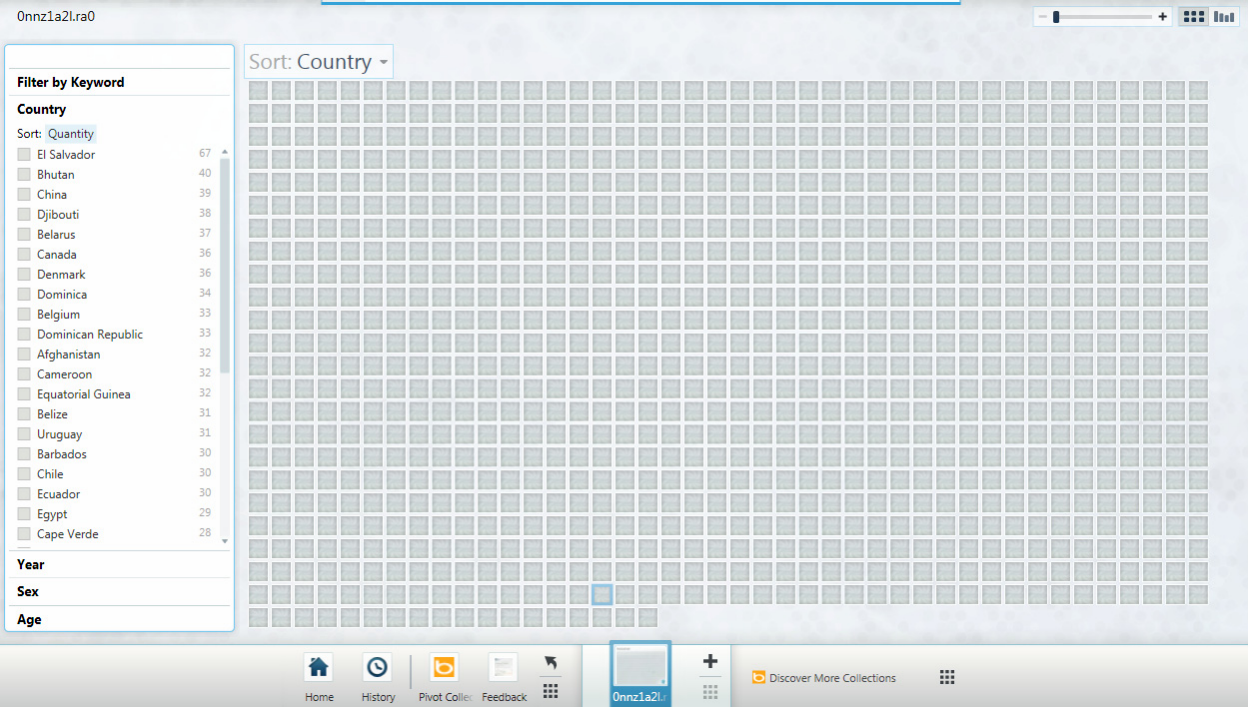
**Quick Preview of the Pivot collection**. The Quick Preview function will show the data classification but will not display the collection images.

**Figure 8:**
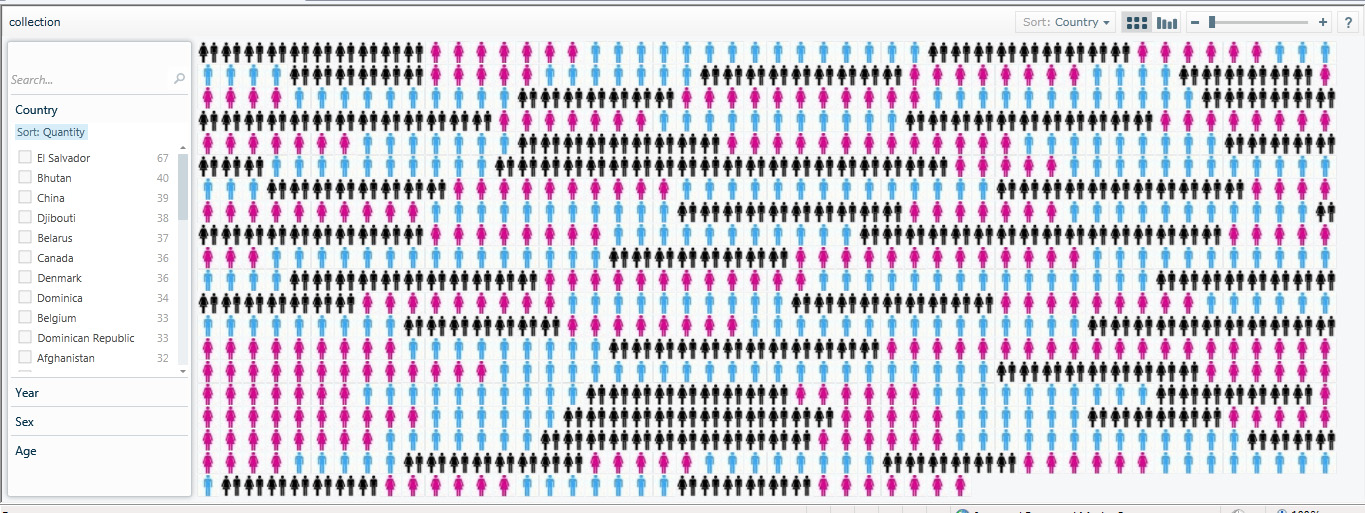
**The published Pivot collection using the WHO mortality sample dataset**.

- **Step 10**: Finally, publish the Pivot collection to the Web (use a Web server such as Apache or IIS) by copying the whole folder generated in Step 9 (when the Pivot collection was published on the local hard drive) to the hosting Web server. Also ensure that collection.html, Silverlight.js, and PivotSimpleDemo.xap files are all copied to the same Web server folder containing the Pivot collection. Edit the collection.html file to point to the URL of the .cxml file on the Web server. Figure 5 shows a snippet of the collection.html file, with a red arrow pointing to the URL of .cxml file.

**Figure 9:**
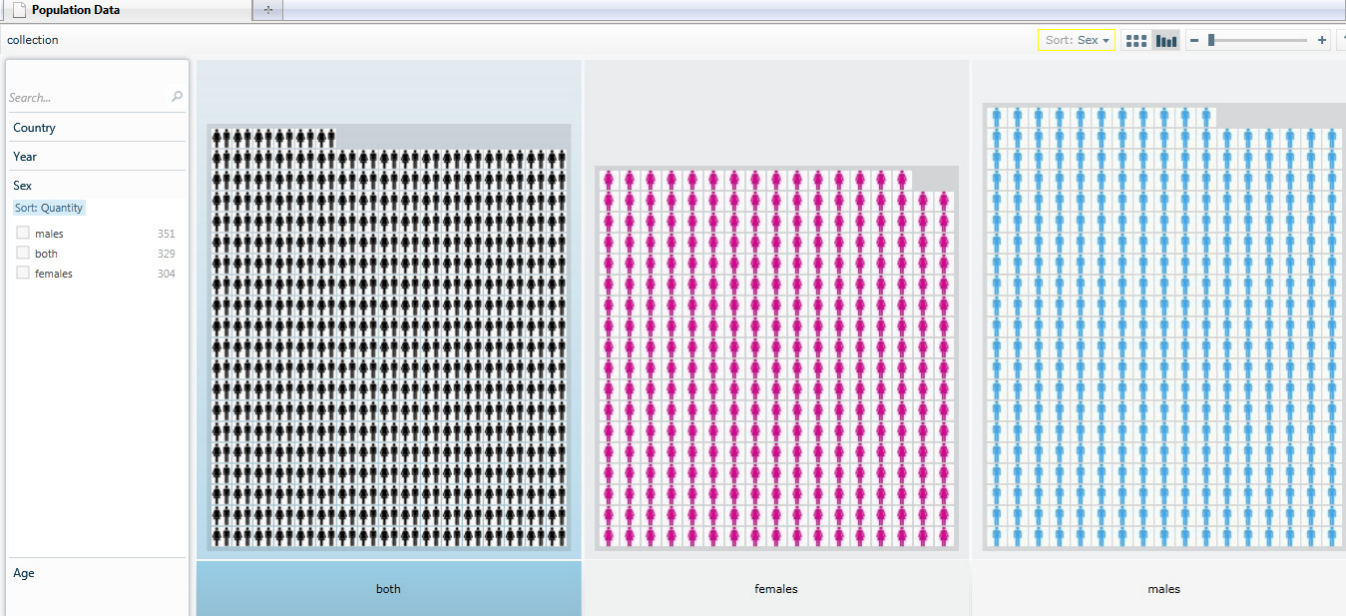
**Classification of the WHO mortality data sample based on sex**. Please note that the current technology demonstrator only uses a small subset of data from the source WHO sheet and, therefore, should not be used to perform any serious country analyses or comparisons, or to draw any mortality data-related scientific conclusions.

**Figure 10:**
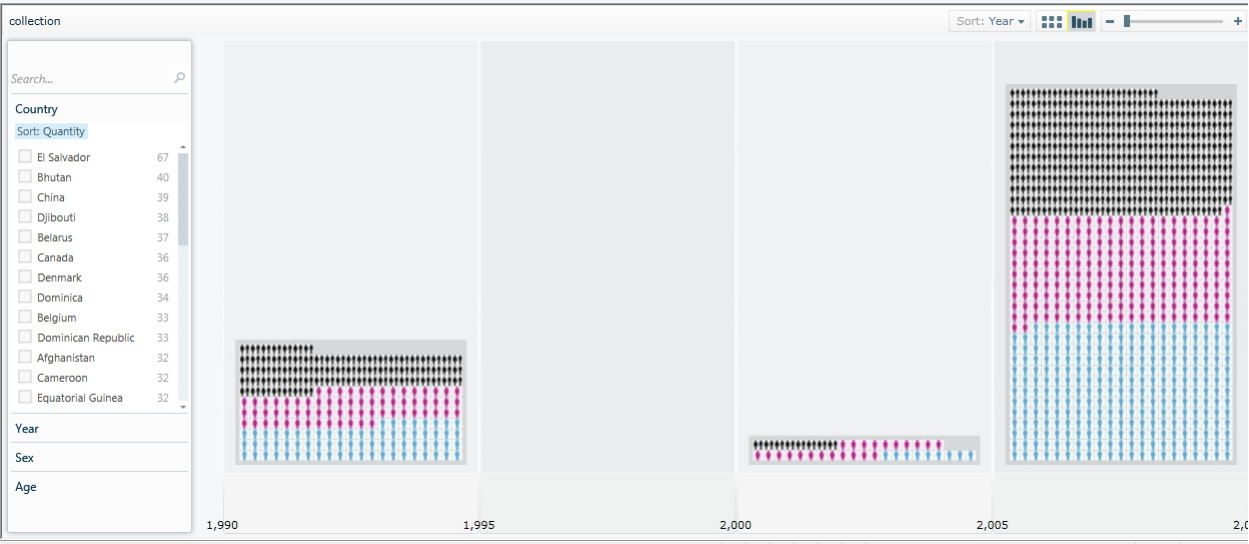
**Classification of the WHO mortality data sample based on year**. The years are wrongly labelled in the current demonstrator; columns in this figure do not represent year ranges but rather individual year figures {1990, 2000, 2008}. Please also note that this technology demonstrator only uses a small subset of data from the source WHO sheet and, therefore, should not be used to perform any serious country analyses or comparisons, or to draw any mortality data-related scientific conclusions.

**Figure 11:**
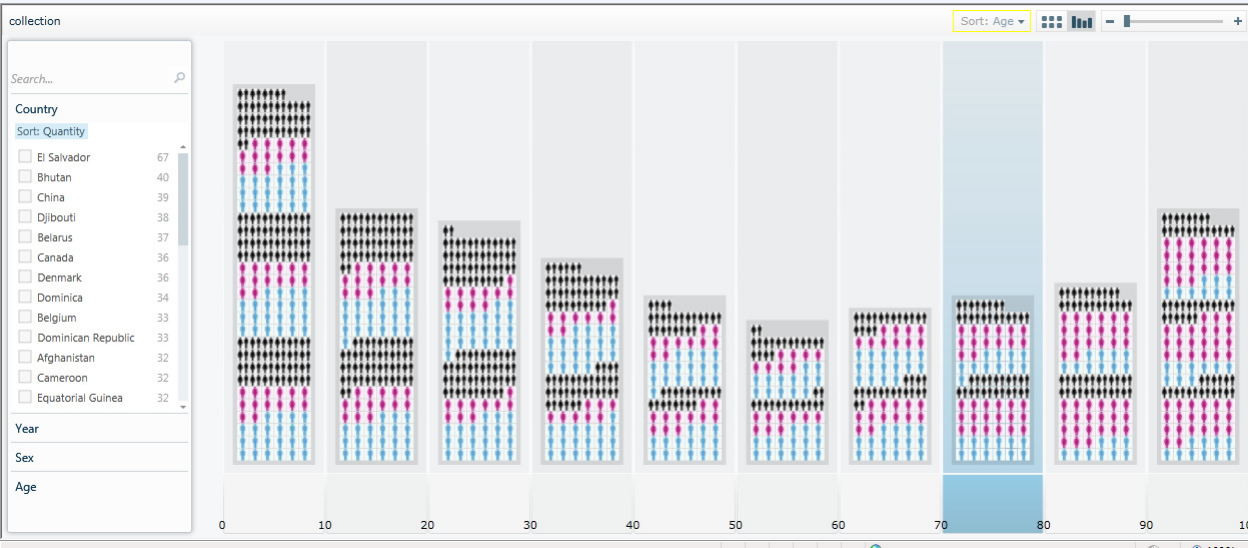
**Classification of the WHO mortality data sample based on age**. Please note that the current technology demonstrator only uses a small subset of data from the source WHO sheet and, therefore, should not be used to perform any serious country analyses or comparisons, or to draw any mortality data-related scientific conclusions.

The demonstrator Pivot collection described in this paper is hosted online at [41]. Readers are encouraged to visit the demonstrator Pivot collection Web site to explore and navigate through the images and data. All the files, modules and library functions used in the demonstrator Pivot collection are also provided in 'Additional file 1'. The Pivot collection of the WHO mortality data shows the classification and clustering of images (representing gender) and data by country, age, sex, and year. There is also a sorting functionality based on those classification criteria. Figure 9 shows the classification of the WHO mortality sample data based on sex. Figure 10 shows another classification of the data by year, while Figure 11 shows the data classified according to age.

Please note that Pivot collections are (at the time of writing) only supported in 32-bit Internet Explorer 7.0 and later or Firefox 3.6 and later; there is no support for 64-bit versions of Internet Explorer, but this might change soon, with the expected release of a 64-bit runtime of Silverlight [42].

## Discussion and conclusions

**Figure 12:**
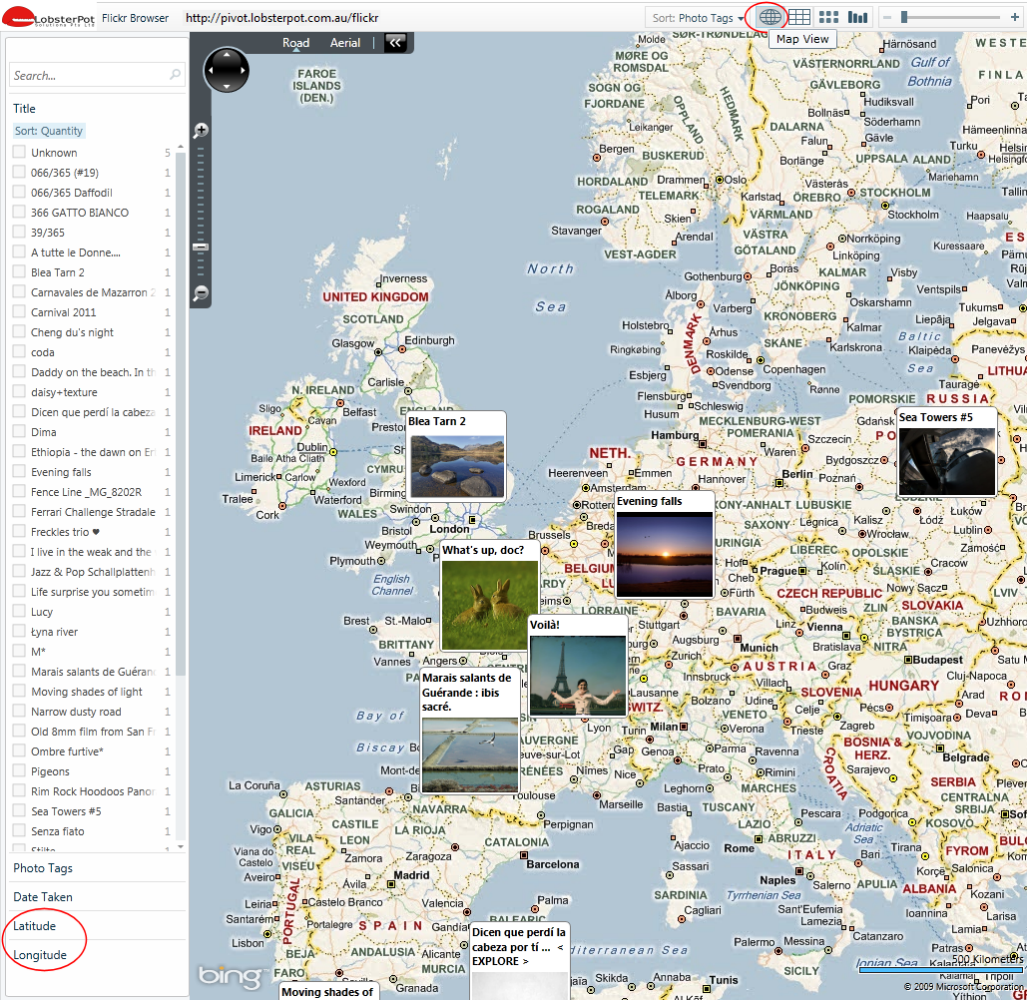
**PivotViewer's Map View example (browsing geo-tagged Flickr images)**.

The Pivot technology demonstrator [41] presented in this paper should not be taken as an accurate representation of any WHO country mortality data (as it is very incomplete and does not include all the data records for the above mentioned 35 countries), but is just meant to demonstrate the potential of the technology and the feasibility (proof of concept) of a full scale WHO mortality data exploration tool using Pivot, based on an idea introduced by Gary Flake in a TED (Technology, Entertainment, Design) video in 2010 [28] for mortality data analysis in Pivot. A full scale tool could also introduce further classifications, e.g., by country income (World Bank classification) and causes of death (*cf. *[43]). Moreover, PivotViewer can be extended to incorporate a Bing Maps layer [44] to map items that have Latitude and Longitude values [45,46] (an example of PivotViewer's Map View is available at [47] - Figure 12).

### Conventional visualisations vs. visual analytics

Visualisations enable not only the intuitive representation and communication of textual and numerical information, but also the highlighting of patterns, trends and relationships in the visualised information that might be difficult to discern using written narrative and numerical forms alone [48]. Visual analytics builds on this, but takes conventional visualisations many steps further by focusing on what a user wants to know (user > question via interactive visual interfaces > query data > data transformation > presentation), rather than merely on what data are available (data > data transformation > presentation). Indeed, the goal of visual analytics is to facilitate the discourse between the user and the data by providing dynamic displays and versatile visual interaction opportunities with the data that can support parallel lines of thought, analytical reasoning, and the exploration of data from multiple user-customisable aspects [17].

### Collaborative and participatory aspects on the Social Web ("Web 2.0")

The rise of Web-based geospatial information visualisation has opened up many powerful opportunities. Web-based visualisations can potentially be combined into visualisation 'mash-ups' and multi-panel analytics dashboards with reusable components. The social and collaborative aspects of visualisation also seem to have taken on new importance on the Web [49]. A number of options exist today for users to aggregate, present, and share these visualisations and analytical artefacts on their own Web sites, in online or e-mail discussions, or in social applications such as blogs, Facebook, etc. (see, for example, Figure 1). Furthermore, some research groups have explored the collaborative and participatory development of Web-based information presentation within the field of visual analytics [50].

### Capturing and documenting analytical artefacts

The ability to document and share analytical artefacts is considered one of the essential requirements in geospatial visual analytics software. Andrienko and Andrienko [12] highlight the importance of the software being able to adequately capture and save analytical observations and artefacts, as well as their spatio-temporal references. There needs to be an explicit and easily manageable representation of the knowledge gained from an analysis; of how the knowledge was obtained (observations, inferences, involvement of background knowledge and additional information from other sources); of any associated assumptions, confidences, conditional judgements, inconsistencies, competing hypotheses, alternative interpretations; and of any relations between the collected analytical artefacts. The ideal tool should be able to capture and store all these details in a knowledgebase without disrupting the analytical process by the user. The knowledgebase should be made browsable and searchable, as well as editable and updatable by authorised users, who can also use it to produce reports about a given analysis and explanations of conclusions made [12].

### Scalability challenges

Andrienko and Andrienko [12] also mention the scalability challenges that need to be addressed in geospatial visual analytics tools, including information scalability (capability to extract relevant information from massive data streams), visual scalability (capability to effectively display massive datasets), human scalability (scaling gracefully from single users to collaborative environments), software scalability (capability of a software system to interactively manipulate large datasets), and display scalability (effective use of everything from wall-sized to phone-sized displays). Yuan *et al. *[15] describe a visual analytics exercise on a large display wall consisting of 32 tiled 32-inch LCD (Liquid Crystal Display) panels, while Pattah *et al. *[51] discuss some techniques that can be used to overcome display limitations on small form factor mobile devices.

While the practical demonstration described in this paper might not fully address all these requirements and challenges, we hope it could still serve as a good introduction to the subject of geospatial visual analytics and stimulate wider adoption of these technologies to empower users at all levels in the public health and healthcare sectors, as well as ordinary citizens [52], to make the most out of the massive and ever-growing amounts of data that are available to us today.

## Competing interests

The authors declare that they have no competing interests.

## Authors' contributions

MNKB conceived and drafted the manuscript with contributions from TV, MNA, VRN, and EK. MNKB also conducted the literature review, wrote all of the Background and Discussion sections, conceived the demonstration exercise idea (inspired by Gary Flake's presentation at [28]), and selected the WHO mortality dataset for it. MNA executed the demonstration in Microsoft Pivot and provided the code in 'Additional file 1'. All authors read and approved the final manuscript.

## Supplementary Material

Additional file 1 **Microsoft Pivot code for the WHO mortality data collection demonstration**. Zip archive containing the Collection XML (CXML) file and other files, modules and library functions used to deploy the WHO mortality data Pivot collection described in this paper. Click here for file
