# Supplementary material for: Web GIS in practice IX: a demonstration of geospatial visual analytics using Microsoft Live Labs Pivot technology and WHO mortality data
Source: Int J Health Geogr. 2011 Mar 16;10:19. doi: 10.1186/1476-072X-10-19 (PMC3068070; doi:10.1186/1476-072X-10-19)
Supplement: Additional file 1 — Microsoft Pivot code for the WHO mortality data collection demonstration. Zip archive containing the Collection XML (CXML) file and other files, modules and library functions used to deploy the WHO mortality data Pivot collection described in this paper. [file 1476-072X-10-19-S1.ZIP › demodata/collection.html]

Population Data
